# Polymeric Nanoparticles for Drug Delivery: Recent Developments and Future Prospects

**DOI:** 10.3390/nano10071403

**Published:** 2020-07-19

**Authors:** Belén Begines, Tamara Ortiz, María Pérez-Aranda, Guillermo Martínez, Manuel Merinero, Federico Argüelles-Arias, Ana Alcudia

**Affiliations:** 1 Department of Organic and Medicinal Chemistry, Faculty of Pharmacy, University of Seville, 41012 Seville, Spain; mariapar89@gmail.com (M.P.-A.); mtnezmun@gmail.com (G.M.); lolo191995@gmail.com (M.M.); 2 Department of Normal and Pathological Cytology and Histology, Faculty of Medicine, University of Seville, 41009 Seville, Spain; tamara.ortiz.cerda@gmail.com; 3 Department of Microbiology and Parasitology, Faculty of Pharmacy, University of Seville, 41012 Seville, Spain; 4 Department of Medicine, Faculty of Medicine, University of Seville, 41009 Seville, Spain; farguelles1@us.es; 5 Department of Gastroenterology, University Hospital Virgen Macarena, University of Seville, 41009 Seville, Spain

**Keywords:** nanoparticles, nanocarriers, polymeric materials, drug-delivery systems, ocular delivery, cancer diagnosis, cancer drug-delivery systems, nutraceuticals

## Abstract

The complexity of some diseases—as well as the inherent toxicity of certain drugs—has led to an increasing interest in the development and optimization of drug-delivery systems. Polymeric nanoparticles stand out as a key tool to improve drug bioavailability or specific delivery at the site of action. The versatility of polymers makes them potentially ideal for fulfilling the requirements of each particular drug-delivery system. In this review, a summary of the state-of-the-art panorama of polymeric nanoparticles as drug-delivery systems has been conducted, focusing mainly on those applications in which the corresponding disease involves an important morbidity, a considerable reduction in the life quality of patients—or even a high mortality. A revision of the use of polymeric nanoparticles for ocular drug delivery, for cancer diagnosis and treatment, as well as nutraceutical delivery, was carried out, and a short discussion about future prospects of these systems is included.

## 1. Introduction

**Figure 1:**
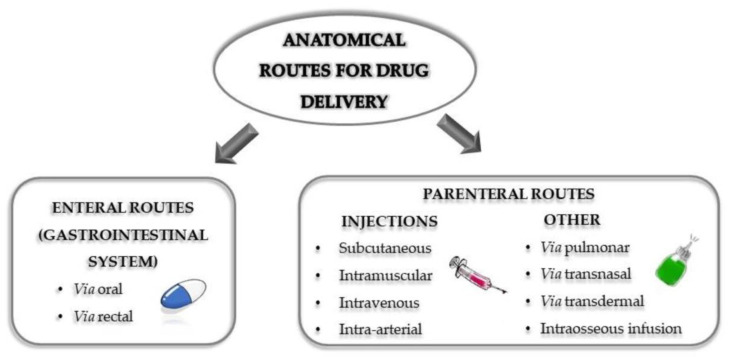
Classification of the different anatomic routes for drug delivery.

The complexity of certain diseases and the toxicity associated with some treatments increasingly demand novel routes for drug delivery. A drug-delivery system (DDS) is a formulation or device that allows the introduction of active ingredients into the body in order to improve not only their efficacy, but also their safety, by controlling the drug amount, time and release in the site of action, crossing the biologic membranes to get to the therapeutic target [1]. This includes not only therapeutic drug administration methods, but also the use of vectors to facilitate their application and diffusion into the human body. In fact, different combinations of vectors and active ingredients may allow a wide range of possibilities for personalization, depending on particular diseases and patients. The routes used to administer and deliver active substances to their target tissue are a relevant factor when treating a disease [2]. These routes may have different effects depending on how they are applied. The administration is normally systemic. Occasionally, due to the severity of the disease or the toxicity inherent to the drug, it must be applied directly to the affected organ. Figure 1 shows the different anatomic routes of administration for drug delivery currently available. All delivery routes present inconveniences when delivering a formulation. As previously mentioned, the potential toxicity inherent to active ingredients or to the high dosage needed to achieve pharmacological effect, is a common disadvantage displayed by the systemic administration routes. The oral pathway of administration, for instance, limits the use of pH-resistant or highly hydrophilic drugs to ensure the required absorption by the intestinal epithelium cells. Likewise, the invasive nature of injections was associated with a high risk of infection.

As stated above, to minimize the risks and disadvantages associated with traditional administration routes, DDSs are becoming increasingly more sophisticated [3], focusing on a better controlled release, maintaining therapeutic efficacy and the active ingredient targeting to the specific site of action, thus avoiding systemic release of the active substance. In this sense, nanotechnology is gaining high relevance, as it could potentially solve some of the issues associated with the above-mentioned traditional administration routes. The bioavailability refers to the portion of the bioactive compound absorbed in the body entering systemic circulation and performing functions. In general, nanoparticles (NPs) could be optimized to improve the drug bioavailability, either by increasing their absorption through enhanced solubility or by facilitating their passage through the biologic membranes [4]. Drug release could also be controlled and maintained at therapeutic levels, by adjusting the composition of the nanoparticulate system. They could even facilitate the combined therapy by the incorporation of more than one active ingredient. The progress in biologic therapies or immunotherapies has been promoted by the advances in nanotechnology, due to the fact that it allows a better administration of gen- or protein-based drugs. Functionalization of the NPs allows the recognition of the specific site of action, avoiding high systemic concentrations and reducing side effects. This property has been very useful in the diagnosis field by combining the specific targeting with the transport and release of a contrast agent [5].

According to the previously described characteristics of a nanoparticulate delivery structure, investigations about the use of different materials as nanocarrier precursors are an essential requirement for the improvement of the applicability and results achieved by these systems. These precursors should meet some requisites such as biocompatibility, biodegradability and non-immunogenicity [6]. Polymers are macromolecules formed by the covalent union of one or different sort of units, named monomers, to constitute a linear or branched chain. These monomers may possess any structure, as long as they have at least two functional groups where they can react with another monomer. Ideally, selecting the right kind of monomer/s, a polymer could be prepared to attain specific properties. Polymers are not only a special type of material that may encompass all the above-mentioned characteristics, but also, the great synthetic versatility they exhibit allows the researcher to customize them according to the requirements or final aims. In order to accomplish certain properties, polymeric tailoring could be carried out directly on biopolymers by chemical derivatization [7,8]. Another option is the preparation of synthetic polymers from their corresponding monomers which can lead to a large range of structures and applications [9,10,11,12]. These are the reasons why polymeric materials are gaining great relevance in nanotechnology in general and are being used as NP precursors for DDSs.

When considering the preparation of polymeric NPs, the use of surfactants may be a requirement. Surfactants are amphiphilic organic molecules that can self-assemble in solution. Most used surfactants are composed by a hydrocarbon chain (hydrophobic section) bound to an ionic functional group (forming the so-called cationic surfactants, such as benzalkonium chloride or tetramethylammonium hydroxide or anionic surfactants, like docusate or sodium laurate). Non-ionic surfactants can also be found, in which the amphiphilic character is generated by the union of hydrophobic and hydrophilic molecules (e.g., ethoxylated amines, alkyl and nonyl-phenol ethoxylates) [13]. Low molecular weight polymers could act as surfactants too, specially block copolymers (e.g., Pluronic F127 or Pluronic P123) [14]. In general, they are commonly included in the nanocarrier formulation as stabilizer agents and may be crucial to obtain a well-structured nanosystem, stabilizing the dispersion during nanoemulsion procedures. Some of the advantages of the stabilizers are to decrease the surface tension of NPs and increase affinity with lipidic structures [15]. Some surfactants have also demonstrated a significant reduction of the mean NPs diameter and also a double action as a cryoprotectant agent [16]. Studies of pharmacokinetics and biodistribution showed increased retention of the drug in the body and accumulation in the target tissue, prolonged time in the blood circulation along with a decreasing nephrotoxicity, hepatotoxicity, lower cardiovascular effects and reduced uptake of macrophage when surfactant surface-modified NP systems are used [17,18]. Multidrug resistance (MDR) mediated by the human ATP-binding Cassette (ABC) transporter superfamily such as *P*-glycoprotein (*P*-gp/ABCB1), multidrug resistance-associated protein 2 (MRP2/ABCC2) and breast cancer resistance protein (BCRP/ABCG2) have been recognized as the main obstacle against efficacy towards multiple chemotherapeutic agents [19]. Both organic and inorganic NPs have been demonstrated to inhibit the MDR. The effects of organic NPs are caused by several excipients, such as surfactants and polymers [20].

This review focuses on the recent advances of the most promising applications of nanoparticulate polymeric formulations as nanocarriers, considering principally those used to treat diseases with a considerable morbidity, a notable reduction in the patient’s quality of life or even an important mortality.

## 2. Polymeric Nanocarriers for Ocular Drug Delivery

**Figure 2:**
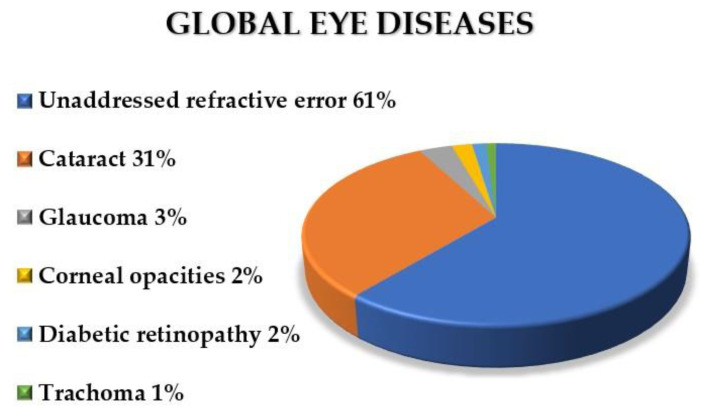
Incidence rate of the most conventional eye diseases, according to the WHO (2019) [21].

In 2019, the World Health Organization (WHO) estimated that at least 2.2 billion people had vision impairment or blindness, of whom, at least one billion cases could have been prevented with the appropriate treatment [21]. Figure 2 shows the incidence rate of the most conventional eye diseases and their corresponding proportions, throughout the world. It is evident that almost half of global blindness or vision impairment could be avoided with the correct treatment. Developing novel and efficient mechanisms for ocular therapy is a current need.

Drug delivery to the eye entails great challenges due to the actual anatomy and physiology of the organ. Two areas can be differentiated: anterior and posterior segments. The anterior segment—composed of aqueous humor, conjunctiva, cornea, iris, ciliary body and lens—is located at the front of the eye, which is readily accessible, making topical instillation of eyedrops the standard method for drug administration [22]. The posterior segment—composed of the choroid, neural retina, optic nerve, retinal pigment epithelium, sclera and vitreous humor—suffers from a low bioavailability at the site of action, due to the reduced period of time that the drug remains inside the ocular globe. Lacrimation, reflex blinking, tear-film turnover or nasolacrimal duct drainage cause a fast elimination of the drug-containing eyedrops. Formulations also need to penetrate different barriers before reaching the posterior segment. This drastically reduces the number of drugs that can achieve therapeutic effects to corticosteroids and cyclooxygenase inhibitors of higher potency [23]. Another option for drug delivery is the administration via systemic route. The presence of the blood retinal barrier drastically reduces the drug access, thus requiring a high dose to reach therapeutic effects. This, in turn, may cause systemic side effects [24]. An alternative for achieving adequate levels of active substance in the action site is directly injecting the drug. This process is associated with high costs, low patient adherence to treatment and elevated risk of injection-related complications. Implementation of intravitreal implants can also be an option for a sustained drug delivery in the posterior segment of the ocular globe, but as in the previous case, it requires numerous injections or even surgery, with the consequent risks [25]. To overcome these limitations, nanotechnological systems for ocular therapy are currently gaining high significance, especially those derived from biodegradable polymers. These systems are mainly designed to achieve an effective dose in the action site, either by improved formulation solubility properties, enhanced bioavailability, targeted delivery, sustained release or a lengthier shelf life [26]. Although different nanosystems for drug delivery to the eye can be found in literature, for example, liposomes [27], nanoemulsion [28], nanodiamonds [29], nanocrystals [30] or inorganic NPs [31], this review focuses on polymer-derived NPs. Some examples of the most recent research regarding polymeric nanoparticulate materials for ocular drug delivery, including micelles, dendrimers, cyclodextrins and polymeric vesicles, will be explored, all of them administered via ophthalmic drops and intraocular injections.

### 2.1. Micelle Nanocarriers for Ocular Delivery

Most commonly used polymers for the synthesis of micelles as DDSs is the poly(lactic-co-glycolic acid) (PLGA), due to its high biocompatibility and biodegradability. It was approved for clinical use in 1989, by the US Food and Drug Administration (FDA) [32]. It has been tested for the sustained delivery of different drugs formulated with polyvinyl alcohol (PVA). Varshochian et al. [33] used the PLGA/PVA system to prepare bevacizumab-loaded micelles for the treatment of ocular neovascularization. This molecule has widely demonstrated its effect in the treatment of retinal and choroidal neovascularization, however, its short half-life in vitreous humor requires frequent intravitreal injections. Surprisingly, bevacizumab-containing micelles provided a sustained release, and the drug concentration in vitreous humor endured above 500 ng/mL, the minimum concentration that completely blocks the vascular endothelial growth factor-induced angiogenesis, for about two months. Dexamethasone, a powerful anti-inflammatory, was also delivered in this type of NP. Ryu et al. [34] prepared dry tablets, administered with a specific preocular applicator, containing dexamethasone-loaded micelles incorporated in a alginate matrix. They demonstrated that the use of this system could increase 2.6-fold the ocular drug bioavailability when compared to Maxidex^®^, a dexamethasone-based eyedrop formulation, which is commercially available. PLGA/PVA NPs were also used to deliver fenofibrate, showing therapeutic effects on diabetic retinopathy and neovascular age-related macular degeneration. Qiu et al. [35] proved that the use of these micelles improved retinal dysfunctions, inhibited retinal leukostasis, diminished retinal vascular leakage and regulated the over expression of vascular endothelial growth factor (VEGF) at eight weeks after the application. Lui et al. [36] described a similar PLGA/PVA based on micelles to treat choroidal neovascularization. In this case, the polymer system also included polyethyleneimine (PEI) to incorporate positive charges in the NP, while the medication was a combination of dexamethasone and bevacizumab, which can interact with the positive charges of PEI. These micelles demonstrated a good anti-angiogenic effect and a strong inhibitory effect on VEGF secretion from human umbilical vein endothelial cells (HUVEC). PLGA NPs have also been formulated exchanging PVA polymer for a different hydrophilic agent, such as Tween 80, poloxamer 188, Pluronic F127 or polyvinylpyrrolidone, among others. Salama et al. [37] developed a brinzolamide-loaded micelles for ocular pressure treatment, which is the most important risk factor for the appearance of glaucoma. They prepared a collection of different nanosystems utilizing a combination of two PLGA, with varied molecular weights and Tween 80, poloxamer 188 or Brij^®^ O10. As well as showing compatibility with the ocular tissue, these micelles proved to reduce ocular pressure for up to 10 days, showing that smaller NPs were able to decrease ocular pressure longer than those with higher particle size. Another type of PLGA-based nanosystem was obtained by Pan et al. [38], who used Pluronic F127 as a hydrophilic agent. In this case, dexamethasone, a drug also used for the treatment of immunologic graft rejection, was encapsulated. Results demonstrated that these micelles prevented corneal graft rejection for at least nine weeks when administered weekly in rats. The control group did suffer from rejection, severe corneal edema, opacity and neovascularization in less than four weeks when dexamethasone in solution was applied. In a different approach, Li et al. [39] utilized polyvinylpyrrolidone to develop PLGA NPs containing bevacizumab as medication against age-related macular degeneration. This system showed a sustained drug-release for over 91 days, although this liberation could be adjusted by modifying the drug/polymer ratio.

Another widely used polymer for the preparation of micelles for ocular delivery is chitosan (CH), a natural hydrophilic cationic polysaccharide. This polymer presents excellent mucoadhesion and penetration properties that make it ideal for drug delivery in mucosa and ophthalmic areas. In this sense, Ameeduzzafar et al. [40] prepared CH-based micelles using sodium tripolyphosphate (TPP) as a crosslinking agent, for the delivery of levofloxacin for ocular infection treatment. This system proved to be biocompatible for topical ophthalmic use, to have a retention time in the ocular area longer when compared with a levofloxacin solution and to reduce corneal clearance and naso-lachrymal drainage. Bevacizumab was also encapsulated in this particulate formulation by Badiee et al. [41]. Drug-loaded CH NPs were later embedded in a hyaluronic acid ocular implant. Although in vivo experiments are not reported, in vitro studies displayed a sustained drug release over two months. Silva et al. [42] developed a similar CH/TPP system—but including hyaluronic acid—another mucoadhesive natural polymer that can react with cell-surface receptors, such as CD44. They encapsulated ceftazidime, a very unstable antibiotic, for the treatment of eye infections such as keratitis. Their studies demonstrated that this nanoformulation presented the required physicochemical and pharmaceutical characteristics for topical eye administration. It was also able to preserve the antibacterial activity while having relevant mucoadhesive properties, by interacting with mucin, an essential condition to improve the residence time in the ocular globe. Some authors have used a different crosslinking agent, instead of TPP, such as sodium deoxycholate. For example, Hanafy et al. [43] used a CH/PVA system crosslinked with sodium deoxycholate to embed prednisolone for the treatment of ocular inflammation diseases. Results obtained on female guinea pigs showed that optimized NPs formulation achieved a twofold increase in the prednisolone release after 24 h when compared with the commercial micronized drug loaded gel. Different routes to obtain CH-based micelles have been explored. One of them is the functionalization with lipophilic derivatives of the CH chain through its primary amino groups. Following this concept, Xu et al. [44] designed a novel branched CH, in which stearic acid and valylvaline were introduced on the polymer main chain in different proportions. These polymeric materials were able to self-assemble encapsulating dexamethasone. Thus, NPs demonstrated access to the posterior segment through conjunctival route, showing sustained release and enhanced penetration properties. In vivo tests carried out in male rats and male New Zealand albino rabbits, exhibited similar dexamethasone levels, compared to dexamethasone-loaded hydrogenated castor oil-40/octoxynol-40 NP, a similar system to Cequa, approved by the FDA. Another option is developing hydrophilic–hydrophobic block copolymers including a CH section. In this line, Shi et al. [45] synthetized a cationic CH grafted methoxy poly(ethylene glycol)-poly(ε-caprolactone) (PEG–PCL) for enclosing diclofenac. The polymer amphiphilic character enables a self-assembly to form micelles at the same time that its positive charges can interact with the negatively charged mucin and increase the NP retention time at the site of action. This formulation showed to be nontoxic and to present enhanced penetration and retention of diclofenac compared with the drug commercial eye drops (1.4-fold higher). The diclofenac concentration in the aqueous humor of rabbits was 2.3-fold higher than that achieved when applying the commercial drug eye formulation.

PLGA and CH are probably the most investigated polymers for the preparation of nanoparticulate formulations for ocular drug delivery, albeit different systems have also been tested. For instance, cyclosporine A, a immunosuppressive agent to treat dry eye syndrome, was encapsulated by Yu et al. [46] in a set of micelles based on the methoxy PEG–PCL block copolymer, modifying the proportions of each block. Results displayed that this system could achieve a 4.5-fold increase in retention effect when compared with 0.05% cyclosporine A emulsion. Another example is the NP obtained by Tang et al. [47] who used a mixture of Tween-80 and polyoxyethylene stearate to encapsulate everolimus (40-*O*-(2-hydroxyethyl)-rapamycin), a drug administered for autoimmune diseases, such as autoimmune uveoretinitis, non-infectious uveitis, corneal neovascularization and immune-mediated rejection after corneal transplantation. They also prepared a nanosuspension containing micelles from PVA, poloxamer P407 (an amphiphilic triblock copolymer containing a hydrophobic section of poly(propylene oxide) and two hydrophilic blocks of poly(ethylene oxide)) and hydroxypropyl methylcellulose. In vivo experiments showed that both systems are promising as ophthalmologic drug carriers, although nanosuspension presented higher release, permeability and bioavailability in New Zealand white rabbits’ eyes. Another possibility is the derivatization of the drug to improve its encapsulation inside the micelles. Huang et al. [48] modified triamcinolone acetonide, a drug used for eye inflammation, as its succinated derivative. Together with PEG–PCL–PEG block copolymer, they obtained a nanoformulation that demonstrated higher transcorneal drug permeability in isolated rabbit cornea, when compared to triamcinolone acetonide suspension. Enhanced therapeutic efficacy against endotoxin-induced uveitis in rabbit model was also displayed.

Some authors improved formulation efficacy by combining the use of drug-loaded micelles with specific media to enhance certain properties, mainly oriented to prolong the residence time of the formulation in the ocular globe. Wen et al. [49] prepared a NP-loaded in situ gel as dexamethasone delivery system for ocular inflammation. The drug was contained in lecithin-based micelles while the in situ gel was obtained by a mixture of poloxamers (P188 and P407). This mixture of polymers is liquid at room temperature, for an easier administration as ocular eye drops, but it turns into a gel at temperatures higher than 35 °C. This property increased the formulation residence time in the eye with the consequent increment in the drug delivery duration. Similarly, Hirani et al. [50] developed a thermoreversible gel loaded with PLGA NPs as drug-delivery system of triamcinolone acetonide for the treatment of age-related macular degeneration. Micelles were prepared using a diblock copolymer of PLGA and PEG, while the thermoreversible gel was obtained by optimization of the amounts of NPs suspensions and a PEG–PLGA–PEG triblock copolymer. Using a different approach, Yandrapu et al. [51] optimized a system in which poly(lactic acid) (PLA) NPs, coated with bevacizumab, were embedded in PLGA microparticles using supercritical CO_2_. This technique has the advantage of avoiding the use of organic solvents or sonication for the preparation of the nanosystem, that could denature the quaternary structure of protein drugs, such as the above-mentioned bevacizumab. With this methodology, Yandrapu et al. [51] demonstrated in a rat model, that the delivery of this drug increased from two weeks, when administered in solution, to two months, when applied in the NP/microparticle system.

### 2.2. Dendrimeric Nanocarriers for Ocular Delivery

Although most of the explored polymeric nanocarriers have focused on micelle-based NP systems, dendrimeric structures have also been investigated. The archetypical dendritic polymer for the preparation of nanosystems for drug delivery to the posterior segment of the eye is based on polyamidoamines. Yang et al. [52] examined the potentiality of dendrimers prepared from a PEGylated polyamidoamine and modified with cyclic arginine–glycine–aspartate hexapeptide and penetratin, as drug carriers. They demonstrated that these functionalized NPs were present in the ocular posterior segment after more than 12 h of a non-invasive administration. A dendrimer based on a polyamidoamine was also studied by Lancina et al. [53] In this case, the dendrimeric core was derivatized with a timolol analog, a common drug used for the treatment of ocular hypertension. Their results displayed an intraocular pressure reduction of 30% in normotensive adult Brown Norway male rats, after 30 min of topical application, in addition to the absence of irritation or toxicity after one week of daily administration. Tai et al. [54] investigated the formation of the complex between polyamidoamine dendrimer and hyaluronic acid. This complex was functionalized with penetratin and loaded with antisense oligonucleotides for the treatment of ocular diseases by regulating the expression of target proteins and genes in cells. This system showed to possess an enhanced eye permeability and distribution to the ocular posterior segment, representing a promising formulation for ocular topical administration. Despite the fact that most recent scientific research dealing with dendrimeric NPs has been based on the use of a certain type of polyamidoamine polymer, some other dendrimer structures can be found in literature [55], such as polylysine (PLL) [56] or phosphorous dendrimers [57].

### 2.3. Other Types of Polymeric Nanocarriers for Ocular Delivery

Although they are less utilized for ocular drug administration, cyclodextrins (CDs) and polymeric vesicles (PVs) are another type of polymeric nanocarriers. CDs are a special type of cyclic oligo- or polysaccharide constituted of six or more units of glucose bound by α-1,4 glycosidic bonds [58]. This characteristic configuration entails truncated cone geometry with an outer surface presenting a hydrophilic character and an internal cavity with hydrophobic feature. This makes them a good option as DDS for hydrophobic active ingredients [59]. In addition, the polarity of these systems can be modulated depending on the number of glucose units that form the cyclodextrin and the variety of their substituents. Rodriguez-Aller et al. [60] developed a library of different cyclodextrins as nanocarriers of latanoprost, an insoluble prostaglandin F2a analog used for glaucoma treatment. Results demonstrated that the ideal candidate as DDS of latanoprost was the propylamino-β-CD, which entails an enhanced ocular tolerance and hence, better patient compliance. It showed better drug stability and availability, as well as lower eye irritation, when compared with commercial latanoprost formulation. Jansook et al. [61,62] used γ-cyclodextrin and randomly methylated β-cyclodextrin to enhance solubility of celecoxib, a non-steroidal anti-inflammatory administered for age-related macular degeneration and diabetic retinopathy. By combination of these nanoaggregates with mucoadhesive polymers, such as hydroxypropyl methylcellulose or hyaluronic acid, they obtained eye-drop formulations that demonstrated improvements in drug permeation through transcorneal and transscleral routes, with no cytotoxicity shown. In a similar approach, Lorenzo-Veiga et al. [63] prepared ocular natamycin nanocarriers. Currently, this is the only drug approved for fungal keratitis treatment. They used a combination of Soluplus and Pluronic P103 and α-cyclodextrin to generate a library of micelles and poly(pseudo)rotaxanes containing the drug. The latter were found to be the most promising candidates since they displayed good diffusion, cornea and sclera accumulation and sclera permeability coefficients.

Although vesicles possess mainly a lipidic nature, some polymer-derivatized phospholipids have been used for their preparation. In most cases, the modification was carried out by the introduction of a PEGylated unit in the vesicle outer surface, since they have demonstrated improved penetration levels, better bioavailability and reduced toxicity compared to non-modified vesicles [64]. In this sense, Zorzi et al. [65] developed a PEGylated PV to encapsulate siRNA sequences for the treatment of ocular keratitis caused by *Acanthamoeba*. A combined therapy of siRNA-loaded PV with chlorhexidine led to a 60% reduction in corneal damage caused by this disease in a murine model. In a similar example, PEGylated vesicles were burdened with natamycin by Patil et al. [66], demonstrating a considerably higher in vitro transcorneal permeability when compared to commercial Natacyn^®^.

Table 1 displays a summary of the composition of the previously described polymeric NPs, the drug they encapsulated and the disease for which they are used.

## 3. Polymeric Nanoparticles in Cancer Diagnosis and Imaging

According to the WHO, cancer is the second leading cause of death worldwide, with an estimated 9.6 million deaths in 2018. These data indicate cancer to be one of the diseases with the highest rate of morbidity and mortality nowadays. Finding effective methodologies for early detection, diagnosis and treatment has become a fundamental objective when developing NPs as DDSs [67,68,69]. Ordinary imaging and diagnosis techniques can only detect tumor mass when it is at least one-centimeter in size, being notably difficult to detect cancer at early stages [70]. This is the reason many researchers are currently trying to develop new and smaller composites able to identify malignant cells related to cancer processes, in order to inform medical staff to devise a treatment strategy. Polymeric NPs have thus emerged as an alternative to limit ordinary contrast agents due to their surface modification abilities and their capacity to regulate solubility of the embedded agents in order to enhance imaging of cancerous cells. Some of the following recent investigations that have been consulted involve both therapeutic and diagnostic objectives (known as “theranostic agents”). This section of the review focuses on diagnostic and imaging results and just mentions some of the therapeutic facets.

### 3.1. Gold-Based Polymeric Nanoparticles Used in Cancer Diagnosis

Gold metallic NPs (AuNPs) and their derivatives are the most important investigation topic when describing new composites able to improve diagnosis and imaging techniques. Due to their versatility, they can be used in multiple imaging methods, providing high resolution and low or non-existent toxicity [71]. Computed tomography (CT) is one of the most commonly used diagnosis techniques in cancer imaging, mainly due to its low cost, high imaging resolution and compatibility with all types of patients. Scanning of soft tissues carried out by this technique requires contrast agents absorbing X-ray radiation. AuNPs have generated great interest as these agents, since they are nontoxic and present up to three-fold more efficiency in X-ray absorption than the current iodine-based CT contrasts agents. Other benefits related to AuNPs are the possibilities of designing and modifying their shape, size and surface. Although there are other NPs with a higher capability of X-ray radiation absorption, like bismuth-sulfide NPs, the control of their characteristics and the modification of their surface are more complicated [72,73]. In order to emphasize AuNPs contrast properties, encapsulation of these metallic NPs in polymeric NPs have been tested. Al Zaki et al. [74] designed and optimized polymeric micelles (AuMs) where 1.9-nm-size AuNPs were encapsulated within the hydrophobic core of micelles constructed from amphiphilic copolymer PEG–PCL. Blood pool contrast was obtained for 24 h and enhanced tumor margin delineation was observed, via CT, when AuMs were injected in living mice. Improvements in survival time when radiotherapy was applied were also demonstrated in these animals when treated with AuMs, compared to those which were not. Dedrimeric NPs were also investigated for the stable encapsulation of AuNPs for CT cancer diagnosis. Lin et al. [75] prepared a CD-derived 21-arm star-like triblock copolymer of β-CD-{PCL-poly(2-aminoethyl methacrylate)-poly[PEG methyl ether methacrylate]}. They combined a dendrimeric NP with the use of a CD unit in its nuclei, not only to stabilize AuNP as imaging agents, but also to embed doxorubicin to obtain a theranostic system. In vitro and in vivo experiments demonstrated the high-contrast properties of this system, characteristic of AuNP.

AuNPs can also be utilized in many other bioimaging techniques such as two-photon nonlinear microscopy, to study the binding coefficient between NPs and target cells and their absorption [76]. Single-photon excitation is a similar technique employed in vitro [77,78] to establish AuNPs accumulation in cells cytoplasm. Wang et al. [79] designed biodegradable polymeric NPs based on silica-coated AuNPs for photoacoustic imaging (PAI). This technique allows researchers to obtain images from biologic structures of different shapes and forms, even from organelles. It consists of the generation of wideband ultrasonic waves (called PA waves) due to thermoelastic expansion when a tissue is irradiated by near-infrared (NIR) light, which is absorbed by the target [80]. It is a very reliable technique to be linked to commonly used clinical diagnosing techniques. The gold nanospheres where synthesized, coated with silica, fluorinated and then introduced in a previously synthesized PLGA NP.

### 3.2. Gadolinium Polymeric Nanoparticles (GdNPs) Used in Cancer Diagnosis

Magnetic resonance imaging (MRI) allows three-dimensional high-resolution images to be obtained. It is useful for delimiting morphologic characteristics in tumors without producing ionizing radiation that could be harmful for the patient. This has become one of the best strategies in clinical cancer diagnosis [81]. To optimize this technique, contrast agents are utilized to enhance the variations between the different tissues, by lowering water relaxation parameter values (longitudinal or T1 and transverse or T2). There are many different types of contrast agents, but gadolinium-based materials are the most widely used [82] and mainly those formed by the chelated metal. While gadolinium-chelated complexes are easily eliminated from the organism by the kidneys because of their low molecular weight (<11 nm), if they are too big, they can be phagocytosed by macrophage cells (>200 nm) [83,84]. Nanotechnology has tried to overcome this inconvenience by designing new gadolinium-based contrast agents with enhanced imaging time, contrast effect and lowered toxicity, as well as granting passive targeting properties [85]. In order to modulate these characteristics, NP surface modification and full size control is necessary [86]. Some investigations have allowed enhancing of imaging by targeting key elements present in cancer cells, such as overexpressed surface proteins. To this end, Liu et al. [87] synthesized a novel multifunctional polymeric GdNPs-based contrast agent (Anti-VEGF PLA–PEG–PLL–GdNP). These nanoparticulate systems were designed with anti-VEGF antibody, which facilitates delivery to cancer cells in hepatocellular carcinoma (HCC) in order to improve its detection in early phases. Obtained NPs were 70–80 nm-sized, preventing them from being easily eliminated from the body. They managed to increase tumor area imaging time significantly in comparison with control substances. In a different approach, polymerization-induced self-assembly (PISA), a synthesis method used in organic chemistry, was applied by Esser et al. [88] to obtain polymeric NPs including Gd ions. The corresponding amphiphilic triblock copolymer poly(glycidyl methacrylate)–block–poly(oligoethylene glycol methyl methacrylate)–block–polystyrene was prepared, which, after self-assembly into the corresponding NPs, was further functionalized with Gd^3+^ chelates. Depending on the polymer composition (proportion of each block), the NP shape and size could be modulated. MRI contrast efficiency was also characterized, compared and classified in terms of size and shape, demonstrating that filomicelles were the most promising candidates as MRI contrast agents.

Gadolinium has also been used as an imaging platform in PAI technique. The great depth penetration that NIR light reaches, makes NIR-light-absorbing materials (650–900 nm) such as organic materials, the ideal candidates for this technique [89,90], even if they are optically unstable. Gadolinium-based agents could overcome this issue, enhancing both imaging time and resolution. Developing polymeric GdNPs where Gd-complexes can be attached and immobilized in macromolecules [91,92,93], red blood cells [94], monoclonal antibodies [95], etc., is a tedious and complicated process. Hu et al. [96] detailed a synthesis pathway to obtain a poly(isobutylene-*alt*-maleic anhydride) (PMA) framework pendent with perylene-3,4,9,10-tetracarboxylic diimide derivatives and PEG, able to self-assemble by ultrasound, to which Gd^3+^ are easily attached. The optimal characteristics of these systems for being used in living organisms was demonstrated when they were injected into mice; excellent biocompatibility and photostability, good water solubility, low toxicity, strong PA signal intensity and a good performance as contrast agents and their ability to passively accumulate in tumors by enhanced permeability and retention effect. Photothermal in vivo treatment improvement was also observed, due to strong NIR optical absorbance and perfect tumor ablation properties, along with the absence of apparent toxic side effects in normal tissues. Wu et al. [97] also described GdNPs specifically designed for MRI/CT/PAI guided photothermal therapy, whose composition was Gd–PEG-coated Bi. These NPs absorb NIR light and transform it into heat, increasing the temperature to 40 °C and producing the in vivo tumor ablation as well as its eradication.

### 3.3. Perfluorocarbons Polymeric Nanoparticles (PFCNPs) Used in Cancer Diagnosis

Perfluorocarbons (PFCs) are molecules whose structure is similar to common organic compounds (e.g., alkanes). The difference between PFCs and regular organic compounds is that every hydrogen atom is replaced by fluorine (^19^F) in PFCs, the most electronegative element in the Periodic Table. This exchange grants new and interesting properties that can be useful for medical applications. Nuclear magnetic resonance (NMR) is usually based on the ^1^H signal from the water of the body’s tissues and mobile hydrocarbon compounds. There are also other nuclei such as ^19^ F [98,99], which can be used in this technique to improve diagnosis and imaging effects. Unlike hydrogen atoms, most of the fluorine found naturally in the organism are located in bone structures, which as solid structures, restrict fluorine signal for MRI assays [100]. One of the major problems connected with the use of PFCs, is their solubility, due to the fact that they have high hydrophobicity. Research and development of new systems able to load contrast agents and enhance their biodistribution, has led to the design of nanoparticulate systems which raise their imaging effects. Kristen Wek [101] designed and characterized a polymeric NP containing fluorine compounds for enhanced NMR effect and passive targeting using a described copolymer [102], obtained from polyethylene glycol methyl ether methacrylate (PEGMEMA) and trifluoroethyl methacrylate (TFEMA) monomers with an azide functional group. NPs were synthesized through atom transfer radical polymerization (ATRP) in order to obtain a small polydispersity index and provide precise molecular weights and sizes. This system also showed passive diffusion into tumors and irrelevant ^19^F NMR signal alteration. In a similar approach, Pisani et al. [103] synthesized polymeric nanoparticles containing liquid PFCs which were sensitive to ultrasound imaging. They synthesized a single core of liquid PFCs and a homogeneous PLGA–PVA polymeric shell, in order to increase the solubility using a variation of the regular emulsion-evaporation methodology. Perfluorooctyl bromide (PFOB), perfluorodecalin (PFD), and perfluorohexane (PFH) polymeric NPs were successfully synthesized and PFOB nanomaterials were characterized and described as nontoxic. In a posterior research work, Giraudeau et al. [104] carried out further investigations and compared these PFOB NPs with free PFOB in several assays, obtaining promising results. Surface functionalization is also used when synthesizing these NPs in order to achieve higher performance levels. Diou et al. [105] added RGD (arginine-glycine-aspartic acid) peptide, commonly considered for active tumor targeting, to the surface of PEGylated polyester nanocapsules of PFOB by pre- and post-functionalization strategies. They were tested in vivo in mice bearing CT26 tumors by ^19^F MRI, showing very interesting results.

### 3.4. Other Nanoparticles Used in Cancer Diagnosis

Although the above-mentioned NPs are the most common nanoparticulate systems currently used for cancer diagnosis, there are others which are under investigation. These are mainly based on different absorbing compounds or on a synergistic union among techniques and/or contrast agents in one single NP. Liopo et al. [106] described the synthesis pathway and characterization of PEGylated biodegradable melanin-like nanoparticles (MNP–PEG) and their properties used in photoacoustic tomography. MNP–PEG demonstrated biocompatibility with human MCF-7 and 3T3 cells and they remained stable in biologic medium for at least eight weeks. Belletti et al. [107] developed a synergy by joining two nanometric concepts, NPs and quantum dots (QDs). Their work was based on the concept that curcumin provokes apoptosis in primary effusion lymphoma (PEL) cells. However, this agent has a very low efficiency rate in this type of cancer treatment, due to its low solubility and consequently, low bioavailability. Encapsulating it in PLGA, NPs enhanced these characteristics and improved the amount of curcumin that was retained by the organism. QDs were also attached to the NPs surface as imaging agents, obtaining a theranostic application. The combination of two different metals in the particulate formulation was also explored. Another example was proposed by Zhou et al. [108] who combined Gd and Au advantages as contrast agents in MR and CT, respectively, to create new imaging agents for targeted dual mode tumor CT/MR imaging in vivo. In this research, PEI modified with folic acid and Gd chelators were used as a matrix to synthesize AuNPs. These systems were then complexed with Gd. Folic acid-targeted PEI-entrapped AuNPs loaded with Gd were characterized, showing good quality properties for in vivo applications: 3.0 nm size Au core, good water dispersion and nontoxicity. Regarding their imaging capabilities, a good X-ray absorption signal, higher than some other commercial contrast agents, and a reasonable r_1_ relaxivity rate were shown, making them ideal candidates for dual mode nanoprobe use for targeted tumor CT/MR imaging in vivo. McQuade et al. [109] established a nanoplatform for theranostic purposes based on gold and superparamagnetic iron oxide NPs (SPIONPs) entrapped within a polymeric micelle, where amphiphilic diblock copolymer PEG–PCL acted as the polymer barrier in a similar assay as the aforementioned work by Al Zaki et al. [74]. On a higher level of complexity, Topete et al. [110] designed polymeric–gold nanohybrids to target multimodal theranostic agents, which are useful in optical and magnetic resonance. These folic acid-functionalized, doxorubicin/SPIONPs-loaded PLGA–Au porous shell NPs were tested in vitro in a human cervical cancer cell line in order to determine their physicochemical characteristics, cellular uptake and theranostic potential. They also reported an improvement in cellular uptake by applying an external magnetic field that guides the nanosystem to the cancer cells, as well as in targeting due to the folic acid.

Even though they are not considered strictly as polymeric nanoparticles, polymer-modified superparamagnetic iron oxide NPs (also called SPIONPs or Fe_3_O_4_ NPs) are remarkable contrast agents which are used in cancer imaging, thus deserving a special mention in this section [111,112,113]. Among their features, their capability to be surface-modified, in order to add polymeric agents that improve their imaging properties, as well as granting active targeting features, is a very powerful tool used in current investigations on developing new imaging agents [114,115,116,117].

## 4. Polymeric Nanoparticles in Oncologic Treatment

As previously mentioned, cancer has become a leading cause of death in developed countries. In fact, experts claim that over the next 20 years, the incidence of this disease is expected to increase by approximately 70% [118,119]. The classic therapeutic approach to deal with cancer consists of surgery, chemotherapy and radiotherapy. Chemotherapy is the treatment of choice in most cancers, but it does present high toxicity due to the affection of both healthy and cancerous cells [118,119,120]. Nanomedicine, defined as the use of materials in nanometric scale in medicine, offers a more specific alternative. Its main objective in oncology is to transport the drug only to cancer cells selectively in order to improve its efficacy and reduce its toxicity [118]. The potential application of nanomedicine can also provide early detection tools in cancer as well as combination therapies that can result in both better efficacy of treatment and prognosis [119].

### 4.1. Advantages of Nanotechnological Drug-Delivery Systems

Chemotherapeutic agents present several inconveniences, including poor aqueous solubility and nonspecific biodistribution. As a consequence, an inadequate drug concentration at tumors or cancerous cells, as well as toxicity to normal cells and possible development of multiple drug resistance, can frequently occur [121,122]. Nanomedicines have been shown to improve solubility of low soluble drugs and to reduce toxicity by dissolving them in their hydrophobic or hydrophilic compartment. Studies also report they have prolonged plasma half-life and a different biodistribution profile compared to conventional chemotherapy [121]. Their nanometric size, large surface-to-volume ratios and the ability for surface functionalization are crucial factors in their biodistribution in vivo [120]. The improvements noted in comparison to conventional free drug administration consist in improving the therapeutic index of the loaded chemotherapeutic active ingredients, increasing drug efficacy by achieving steady state therapeutic levels over an extended period of time, lowering drug toxicity due to controlled drug release systems and an improvement of solubility and stability [120,121]. Other notorious advantages of nanomedicines are the possibility of surface functionalization, as well as the possible combination of multiple drug-delivery systems to achieve a synergistic therapeutic effect. This field also allows the application of a combination therapy fusing chemotherapeutic and photothermal effects or creating magnetic nanostructures, making NP delivery easier with the application of an external magnetic field [121].

Currently, the nanoparticulate systems approved for their clinical utilization are liposomes, albumin-based NPs, polymeric NPs and inorganic NPs [120]. Some polymer-derived liposomes or even polymer–inorganic NP composites, can be found in literature. This section will focus on the polymeric NPs as DDSs in oncology. In the last decade, biodegradable polymeric NPs have been widely considered as potential drug-delivery systems for their application in oncology. Polymers are the most common materials for constructing NP-based drug carriers. Due to their nature, they make possible the customization of many properties, such as hydrophobicity, molecular weight and biodegradability [119]. In most cases, polymeric NPs are spherical and are constituted of dense matrices. PLA, polyglycolic acid (PGA) and PLGA are the most common synthetic polymers used in these carriers. Natural polymers, such as dextran, gelatin, guar gum, collagen and CH can also be utilized. All of them are completely biocompatible and biodegradable, and have acknowledged degradation curves, making the drug release process of these nanocarriers more accessible to be customized in comparison to other nanoparticulate DDSs [118,119,123]. Moreover, performing nanoparticle-surface modifications by using different types of ligands to the receptors over expressed on the cancer cells, these polymeric NPs can be targeted directly to them [121].

Currently, albumin is being widely used for the preparation of NPs as DDS due to its intrinsic characteristics. It is a very stable, soluble, acidic, non-immunogenic, nontoxic and biodegradable protein. In addition, it has a high conjugation capacity due to its several binding sites and a long half-life (19 days). It accumulates naturally in the tumor environment due to its advantageous incorporation. When albumin interacts with some epithelial cell surface receptors, it is actively transported via transcytosis, a process that includes both endocytosis and exocytosis. One of the most relevant receptors, except in the brain, is glycoprotein 60 (Gp60), which is overexpressed in vascular endothelium cells that compose tumor blood vessels. Along with this receptor, the accumulation of albumin is even more facilitated by SPARC glycoprotein [124]. This protein has a significant homology to Gp60, and its overexpression is related to cancerous processes and the higher rate of binding and subsequent uptake by tumor cells has been described in several tumor model experiments. These facts, united to the enhanced permeability and retention effect (EPR) in solid tumors, which is discussed in detail in this work, allows albumin-based NPs to be directed straight to tumoral cells without the need of active targeting with antibodies or other mechanisms [125,126,127,128].

### 4.2. Challenges Associated with Nanoparticulate Drug-Delivery Systems

Despite their advantages and formidable potential, some inconveniences in their use can be found related to their limited shape, chemistry, wide size distribution agglomeration state and electromagnetic properties that can lead to poor oral bioavailability, instability in circulation and inadequate tissue distribution. The vast majority of polymeric NPs are spherical, yet a wide range of different sizes may be generated during synthesis. Their continual interaction with living cells could trigger a range of adverse effects in humans and animals [121,122]. To resolve these problems, new techniques are under investigation, being particle replication in no wetting templates (PRINT) the most recent approach in this field. PRINT technique concedes the synthesis of uniform polymeric NPs, permitting the customization and stabilization of properties such as shape and size. Therefore, the amount, rate and pathway for the uptake of the encapsulated drug in the formulation could be tailored as well [119].

### 4.3. The Enhanced Permeability and Retention (EPR) Effect

**Figure 3:**
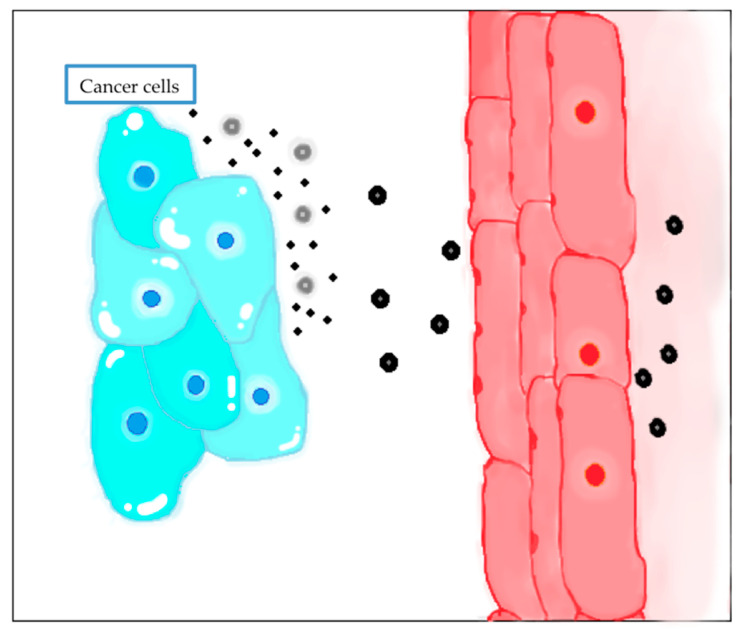
Schematic of the EPR effect: NPs pass through the endothelial fenestrations and reach cancer cells.

The EPR effect is a particular paradox that occurs only in solid tumors, and it is directly related to their pathophysiological and anatomic characteristics, which differ from normal tissues. This effect was first described by Matsumura and Meda in 1986 and has been studied since then. Most solid tumors have abundant, but aberrant, vasculature and poor lymphatic drainage because of the compression of the cancerous cells and the dysfunctional lymphatic angiogenesis [120,129,130,131]. Their blood vessels have an abnormal architecture and produce an excess of a variety of vascular permeability factors, resulting in an increased vascular permeability to ensure the supply of oxygen and nutrients to tumor tissues [129,130]. In addition, it has been demonstrated that the endothelial junctions of tumor blood vessels are larger than normal. Thus, macromolecules larger than 40 KDa, can percolate through these enlarged junctions and accumulate in the interstitial fluid and environment of the tumor. Therefore, the EPR effect depends on the molecular weight of molecules or particles, being only those larger than 40 KDa, which correspond to the limit of renal clearance, the ones that experiment this effect (Figure 3). In addition, these macrostructures remain in the tumor for a considerably long time (several days). The EPR effect has become the principal mechanism to consider in oncologic treatment design. Nanocarriers are designed to take advantage of this EPR effect and accumulate in the tumor environment to achieve the best targeting and therapeutic efficacy [120,131]. Only poorly vascularized tumors, mainly pancreatic, prostatic and liver metastatic experience less EPR effect than other types of cancer. Meda et al. [132] discovered that substances that emulate the effect of vascular mediators involved in EPR effect could enhance it, principally nitric oxide (NO), as well as bradykinin, prostaglandins and VEGF, by facilitating tumor angiogenesis and growth. Recent developments have also demonstrated that another vascular mediator, carbon monoxide (CO), also enhances this EPR effect. It has been demonstrated that this effect was more present when the systolic blood pressure was higher than normal. In order to intensify the EPR effect, blood pressure can be elevated, or NO-releasing and CO-releasing agents can be administered [129,130,132,133]. It is important to mention that, with elevated blood pressure, studies showed that the accumulation of nanocarriers in the tumor was significantly higher and the release of these substances in healthy tissues was lower, due to vasoconstriction and compaction of endothelial junctions, translating in less toxicity [129].

This pathway could indeed be beneficial in order to deliver NPs to the tumor, but it also encounters several challenges. First, an increment of interstitial fluid pressure due to a leak of proteins caused by the tumor growth, supposes a barrier that can block the penetration of NPs inside the tissues. The pressure on the abnormal lymphatic vessels caused by the tumor cells, provokes a considerable reduction in their drainage, contributing to the accumulation and increment of the fluid pressure. Finally, the heterogeneity of the tumor tissues leads to an abnormal distribution of the nanocarriers, as the central part of the tumor is normally less vascularized or necrotic. Indeed, penetration of the NPs to this central area is poor via EPR [120,122,131].

### 4.4. Active Targeting

**Figure 4:**
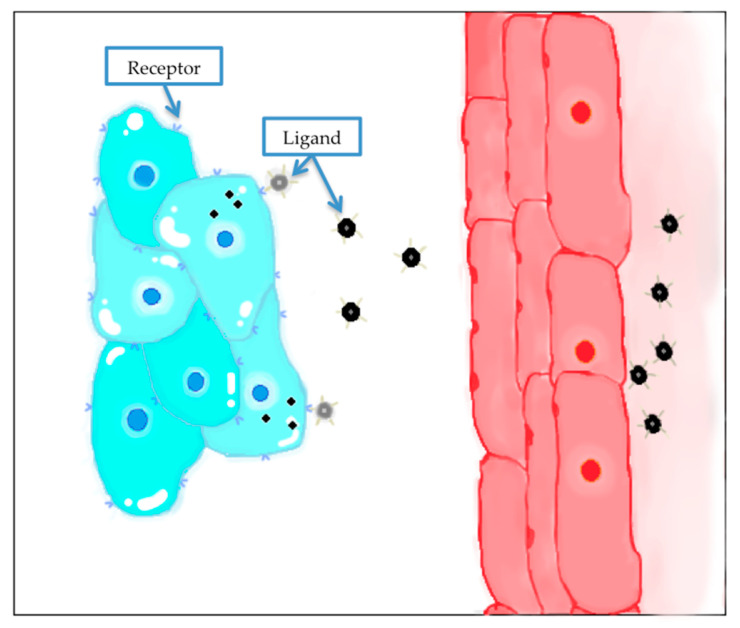
Schematic of the active targeting process: NPs have been functionalized by adding ligands onto their surface that can recognize and bind the receptors in cancer cells.

This method, based on molecular recognition processes, consists of modifying the NPs surface with one or more required moieties to achieve their functionalization and, in consequence, raising the drug concentration in tumor tissues. The most frequent targeting components are monoclonal antibodies and antibody fragments, antigen biding such as fragments and single chain variable fragments (Figure 4). Other molecules that can be utilized are transferrin, enzymes, folic acid and other proteins or peptides. These compounds recognize and bind receptors in the cancerous cells. Normally these ligands are selected depending on which receptor is more overexpressed in tumor cells compared to normal tissues [118,120]. If the selected receptors are internalizing, then the nanocarriers will be transported into the cancerous cells by a specific pathway. In addition, these ligands can be directed to endothelial cells of solid tumors blood vessels to improve the accumulation of nanocarriers in the site of action [134]. Therefore, the purpose of active targeting is to improve the incorporation of the nanocarriers to the cancer cells. Active targeting is a complementary approach to the EPR effect, to improve addressing to the tumor.

Some of the most relevant receptors that are overexpressed in cancer cells are adenosine, transferrin, somatostatin and folate receptors, epidermal growth factor receptor (EGFR), glucose, integrins, chlorotoxin and cytokeratin [135].

Finally, the election of the ligand depends on numerous factors, such as the type of targeted tumor, characteristics of the overexpressed receptors, if internalization process is possible and the proper structure and biodistribution of the nanocarrier [134].

### 4.5. Stimuli-Responsive and Triggered Release Systems

**Figure 5:**
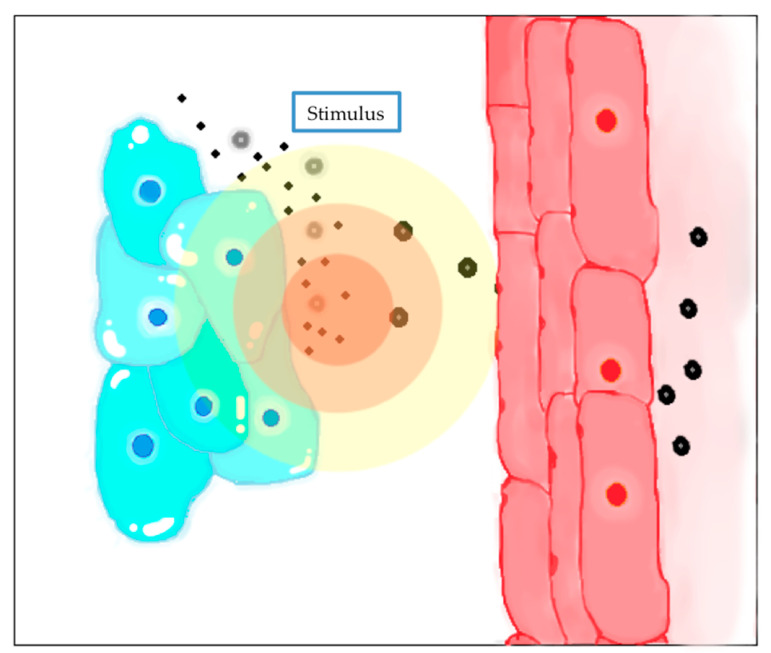
The exposure to a stimulus triggers congregated nanoparticles drug release.

The objective of these systems is the controlled release of antineoplastic drugs provoked by stimuli that develop a change in the nanocarrier (Figure 5). Both internal (changes in pH, redox, ionic strength) and external stimuli (temperature, magnetic fields or light) can trigger the release of drugs [119].

To date, many polymeric NPs have been used in the carriage of antineoplastic drugs like paclitaxel, doxorubicin or camptothecin in many types of cancer. The use of these polymeric NPs can provide improvements in cancer therapy by exploring new routes of administration of some drugs, combining some active substances to potentiate their action or combining with other therapies like gene therapy. Ahmad et al. [136] proposed an enhancement of doxorubicin oral bioavailability through surface modified biodegradable polymeric NPs as an alternative to intravenous administration. They studied drug-loaded PEGylated PLGA NPs pharmacokinetics compared to doxorubicin in Wistar rats. Results showed that NPs had better activities and also higher bioavailability compared to oral drugs. Soma et al. [137] studied the synergistic effect of polyalkylcyanoacrylate (PACA) nanoparticulate formulation of cyclosporin A and doxorubicin compared to only NPs in resistant tumors. Results showed that the combination of both active ingredients were more effective in terms of growth rate inhibition in P388/ADR cells.

There have been numerous examples of the use of polymeric NPs in concrete oncologic diseases. Albumin-bound (nab)-paclitaxel NPs (Abraxane^®^) were approved in 2012 by the US Food and Drug Administration (FDA) for cancer treatment. It has been used since then for the treatment of a large list of cancer including non-small cell lung carcinoma, metastatic breast cancer and pancreatic cancer. These NPs were developed to improve the pharmacokinetics and pharmacodynamics of paclitaxel and also to avoid the toxicities of polyoxyethylated castor oil solvent (Cremophor), used for paclitaxel because of its poor aqueous solubility. In addition, these NPs, in combination with gentamicin, have slightly improved survival rate in advanced and metastatic pancreatic cancer. One of the latest nanoformulation of novel paclitaxel liposome–albumin composite obtained a high encapsulation efficiency of 99.8% [138].

In brain tumors, Cırpanlı et al. [139] studied the activity of camptothecin-loaded cyclodextrin NPs in a rat glioma model. This nanoparticle suspension was injected by convectional enhanced delivery at the same coordinates where the tumor cells were. The use of nanomaterials prevented the drug from hydrolysis and allowed its action. Results showed an improvement of the survival time and determined camptothecin-loaded amphiphilic cyclodextrin nanosystems as an effective nanocarrier. Concurrently, placlitaxel-containing PEG–PLGA NPs coated with AS1411, a DNA aptamer which binds to a protein highly expressed in the surface of cancer and endothelial cells of gliomas, were developed by Guo et al. [140]. Results in vitro and in vivo showed higher tumor growth inhibition compared to placlitaxel-NPs alone and Taxol^®^. In vivo experiments were carried out in Sprague–Dawley (SD) rats, Wistar rats and nude mice and the formulation was administered via the tail vein. Malinovskaya et al. [141] studied the improvement of crossing the blood–brain barrier, which is the principal issue in the therapy of intracranial tumors, using PLGA NPs overcoated with poloxamer 188 for the treatment of glioblastoma in U87 human cells. Hekmatara et al. [142] investigated a system constituted by doxorubicin bound to polysorbate 80, that was in turn coating poly(butyl cyanoacrylate) NPs, in a orthotopic rat 101/8 glioblastoma model, in comparison to doxorubicin in solution, both administered via intravenous injection. The group treated with this nanosystem showed better antitumor effect compared to the control.

Breast cancer is the most prevalent cancer in women, representing an overwhelming 30% of all diagnosed cases of this disease. The notorious diversity in subtypes of breast cancer and their variable response to distinct treatments lead to a great difficulty to develop a universally effective treatment [143]. The use of nanocarriers in the treatment of this type of cancer opens a door to improve their effectiveness. Yuan et al. [144] studied the action of pH-sensitive PEG–PLGA–PGlu (polyglutamic acid) NP embedded with curcumin and doxorubicin in drug resistant cancer stem cells and tumor cells of breast tumors, obtaining good results in mice models. The combination of the use of nanoparticulate systems with photodynamic therapy in breast cancer were investigated by Hu et al. [145]. They developed oxygen-generating theranostic NPs of poly(ε-caprolactone-co-lactide)-*b*-PEG-*b*-poly(ε-caprolactone-co-lactide) with doxorubicin, chlorin e6 and colloidal MnO_2_ to generate oxygen in the tumor environment, relieving tumor hypoxia and improving photodynamic therapy and doxorubicin action. MDR has also been investigated for breast cancer. Shafiei-Iranneja et al. [146] demonstrated that the use of polymeric NPs is a good strategy to combat MDR in doxorubicin-resistant breast cancer (MCF-7/DOX) cells. However, the nanoencapsulation of these NPs together with D-α-tocopheryl polyethylene glycol 1000 succinate (TPGS), a compound used for surface modification of PLGA NPs, has shown a higher cytotoxicity and apoptosis in breast cancer cells [147]. In addition, a higher intracellular drug accumulation and a reduced drug efflux, associated with a decreasing cellular ATP content and an inhibition of *P*-gp activity, have been observed [146]. Additionally, Vitamin E Succinate (VES) that exists as the hydrophobic moiety of TPGS, has also been shown to represent a promising strategy for delivery of doxorubicin into MCF-7/ADR cancerous cells and to revert MDR [148]. Treatment options for triple-negative breast cancer subtype are narrowed down to traditional chemotherapy, surgery and radiation. It is well known that these treatments are not tumor selective and are not very effective, especially when metastatic disease is present. Khanna et al. [143] proposed the use of perlecan-targeted PLA–PEG–maleimide NPs for drug delivery in the treatment of this specific type of breast cancer. The overexpression of a cell surface protein, perlecan (HSPG2), has been recently identified, in this particular type of cancer. This molecule is a large basement membrane protein that is remarkably glycosylated, and it plays a role in binding growth factors. Regarding this fact, researchers have developed two monoclonal antibodies (Clone 6 and AM6) that attach with great affinity to perlecan (HSPG2) present in tumor cells. Indeed, paclitaxel-loaded PLGA NPs were functionalized with these two antibodies using thiol–maleimide chemistry. The antibodies were covalently conjugated to NPs without affecting antibody binding affinity or NP properties. Results of in vitro and in vivo models of triple-negative breast cancer showed that perlecan-targeted NPs improved cell uptake, retention, cytotoxicity in vitro and enhanced tumor growth inhibition in vivo.

Liver cancer has become one of the most frequent cancers and its mortality rate is considerably high, being the third cause of death provoked by oncologic pathologies. Most antineoplastic drugs have high liver toxicity and can trigger severe side effects. Polymeric NPs have been employed as promising carriers for anticancer drugs, not only to improve their efficacy, but also to reduce the appearance of side effects. Zhu et al. [149] synthesized a nanosystem based on a new galactosamine-conjugated polydopamine-modified copolymer (Gal–pD–TPGS–PLA). In vitro cellular uptake and cytotoxicity assay showed that Gal–pD–TPGS–PLA NPs target HepG2 cells via ASGP receptor-mediated recognition and remarkably inhibit cell proliferation. In addition, docetaxel-loaded Gal–pD–TPGS–PLA NPs reduced tumor size more, evidently in vivo, than Taxotere^®^, docetaxel-loaded TPGS-PLA NPs or pD-TPGS-PLA NPs or saline.

Table 2 shows a summary of the nanoparticulate systems as DDS for cancer treatment presented in this review.

Lung cancer is also one of the most prevalent. Hu et al. [150] explored the efficacy of paclitaxel-loaded polymeric NPs combined with circadian chronomodulated chemotherapy. In vitro results showed that this nanosystem exhibits best anti-cancerous activity against A549 cancer lung cells compared to paclitaxel and also determined that the best time of the day to be administered was 15 h after sunrise. In addition, the use of smart PEG-derived polymeric NPs to codeliver paclitaxel and siRNA against survivin gene in lung cancer was proposed by Jin et al. [151]. In vitro results demonstrated that the nanoparticulate formulation presented less toxicity and more antiproliferation effect of paclitaxel on A549 cancer lung cells. In addition, in vivo studies showed accumulation of NPs in the tumor environment and their ability to impede tumor growth. The survival rate was higher because of the-silencing of surviving gene and the action of paclitaxel into tumor cells. The use of inhalable nanocarriers to deliver antineoplastic drugs has been developed recently due to their specific characteristics. These nanocarriers highly associate with drugs and they sustain their release. They can be efficiently transferred into aerosols and remain highly in nebulization state. They also have the capacity to avoid mucociliary clearance as well as respiratory phagocytic mechanisms, thus prolonging the permanence of the antineoplastic drug within the respiratory tract. Polymeric NPs have been extensively used for the aerosol delivery of chemotherapeutics, genes or their combination for lung cancer therapy. Different examples of tested nanoparticulate systems prepared as inhalable polymeric nanocarriers are enumerated in Table 3.

The main inconvenience related to the use of inhalable NPs is that—due to their nano-range size (<0.5 μm)—they could be quickly exhaled even before they reach the site of action. Currently, other methods to improve pulmonary drug delivery are being investigated. Principally, the combination of both microparticles (MPs) and NPs are the main option. MPs permit deeper lung deposition, but they are opsonized by alveolar macrophages. Studies are directed to develop a functional combination of NPs and MPs, where the MPs act as transporters to deeper areas in the lung where NPs could not reach [140]. Although these protocols have an enormous potential, they are not exempted of health risks. First, the deposition of insoluble nano- or micro-carriers can provoke a local inflammatory response and oxidative stress. It has been demonstrated that surface charge of NPs has a significant role in lung toxicity. While anionic biodegradable NPs showed good tolerability, cationic NPs caused toxic effects. On the other hand, NPs can react with the pulmonary surfactant and provoke a quick decrease in the surface tension during compression/expansion cycles, resulting in life-threatening consequences to the patient. The development of safer nanocarriers for pulmonary drug delivery is being intensively researched. Some physicochemical properties are related to the harmful potential, such as particle size, shape, structure, biodegradability and surface charge. Active targeting via surface modification is also being investigated, to enhance NPs accumulation into lung cancer cells via receptor-mediated endocytosis.

The use of carriers for the intracellular delivery of macromolecules of DNA or siRNA for nanoparticle-based gene anticancer therapy is considered the next generation of medicine. Polymeric nanoparticles are one of these nanocarriers. In fact, they can be used in combination with other therapies, for example, the codelivery of chemotherapeutics drugs and small siRNA by using smart polymeric NPs with pH-responsive and PEG-detachable properties, was explored by Jin et al. [151] with promising results. In cancer therapy, the goals of the transmission of genetic material into specific cells can be the correction of the mutation present, RNA interference, trigger the immune response against cancer cells, induce an antiangiogenesis effect, produce cytotoxic proteins or produce enzymes that helps in the activation of some antineoplastic drug. The nanocarriers must be capable to deal with numerous obstacles related to the internalization of the genetic material such as cellular membranes (extra and intracellular), the process of endocytosis and the later breakout from endosome and nucleus. In addition, they must be biocompatible, nontoxic, non-immunogenic and stable and must be able to protect the genetic material from degradation, characteristics that are applicable to polymeric nanoparticles. In the last years, this field has advanced notoriously and has been directed towards the development of multifunctional NPs for cancer treatment and diagnosis. At the moment, the codelivery of chemotherapeutic drugs and genetic material with a synergistic effect is the main subject of study. It is important to highlight that the risks associated with the use of these therapies are not well known and the results obtained so far are not conclusive. In fact, some studies suggest that NPs could interfere with cellular biologic functions, including at genetic levels. Because of that, nanotoxicology is also a developing field, very necessary to a future establishment of regulations and guidelines [174]

## 5. Polymeric Nanoparticles as Nutraceutical Agents

**Figure 6:**
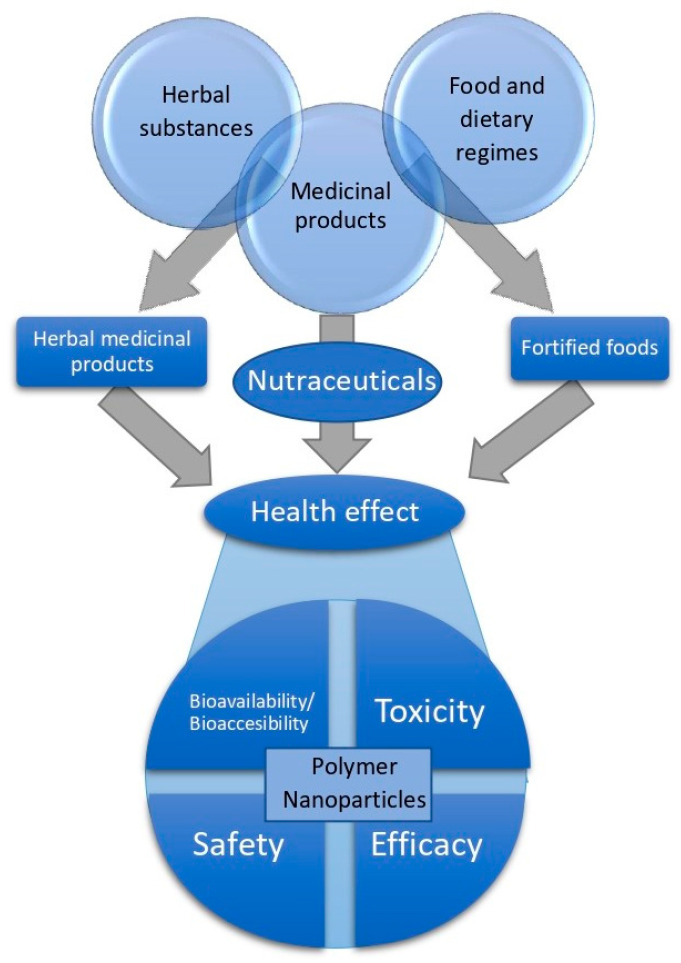
Use of polymeric nanoparticles for nutraceuticals and different bioactive compounds for greater health and medical benefits.

Although there is no official accepted definition of nutraceuticals, they are mostly referred to as pharma-foods, a powerful toolbox to be used as a complement to the diet and before prescribing drugs, in order to improve health and prevent and/or treat pathologic conditions. Subjects could be people who may not yet be eligible for conventional pharmaceutical therapy [175]. There is widespread inconsistency and confusion in the definition of “nutraceuticals”. Substances from similar sources are classified differently, such as plant-derived drugs, for example, digoxin from foxglove leaves is in the group of the medicinal products, while extracts from green tea leaves are regarded as nutraceuticals [176] (Figure 6). Regarding the legislation, in the United States of America, the FDA regulates dietary supplements, which include nutraceuticals, under the Dietary Supplement Health and Education Act of 1994 (DSHEA). In contrast to Canadian regulations, research studies in humans to prove dietary supplement safety/efficacy, are not required by the FDA prior to marketing [177]. The current European regulations consider nutraceuticals as belonging to the same category as food supplements. The Directive 2002/46/EC on food supplements and novel foods, which was recently modified by the new European Parliament and Council Regulation (EU) 2015/2283, defining new foods categories, completes the classification of food supplements, but it still does not mention the term ‘nutraceutical’ [178].

The use of nutraceuticals for several pathologies has been reported. Some nutraceuticals can be used to reduce some of the main cardiovascular risk factors, such as altered blood glucose levels, hypertension and hypercholesterolemia [179]. The most frequently occurring cholesterol-lowering and blood–pressure lowering substances found in nutraceuticals are the following: berberine, beta-glucans, sterols, isoflavones, mono unsaturated fatty acids and monacolin K (also known as lovastatin) from extracts of red yeast rice fermented by *Monascus purpureus* [178,180] or the use of potassium, magnesium, L-arginine, vitamin C, cocoa flavonoids, beetroot juice, coenzyme Q10, melatonin and aged garlic extract [181]. In the case of the glucose metabolism and type 2 diabetes mellitus (T2D) the evidence suggests that increasing omega-3, omega-6 or total polyunsaturated fatty acids (PUFAs) has little or no effect on prevention and treatment of T2D [182], but randomized controlled trials suggest that viscous dietary fiber at a median dose of ~13.1 g/day may offer beneficial effects on glycemic control and, thus, an improved cardiovascular disease risk profile [183]. In addition, vitamins, mainly vitamin C and vitamin D, have been recommended as nutraceuticals to reduce periodontal risks or improve periodontal health [184]. Riboflavin, coenzyme Q10, magnesium, butterbur, feverfew, and ω-3 PUFAs have been recommended for adults with migraine [177]. Nevertheless, the evidence of the efficacy of nutraceuticals for the treatment of pediatric migraine is limited [185]. Many nutraceuticals have been considered useful, not only to treat some pathologies, but also to mitigate disease-related symptoms. In osteoarthritis, a chronic disease, the nutraceuticals may represent promising alternatives for the relief of pain, where the conventional pharmacological approaches to pain relief and joint repair have not always been safe for long term use [186].

### 5.1. Bioavailability and Nanoparticles

As previously mentioned, the bioavailability refers to the portion of the bioactive compound that is absorbed in the body entering systemic circulation and performing functions. In order to determine bioavailability, it is necessary to measure blood plasma levels [187]. There are many animal model and epidemiological studies associated with food supplements or nutraceuticals, indicating their effectiveness and safety, however, the bioavailability is not clear [188,189,190]. The bioavailability of several nutraceuticals depends on many factors, such as dosage, possible interaction with the food matrix, like protein and fibers, the hydrophobicity of the compound, low chemical stability, intestinal first-pass metabolism [178,187,191] and gut microbiota, which can catabolize non-absorbed nutraceuticals and generate metabolic products that can have physiologic effects, and at the same time, prebiotic properties [192]. For example, the bioavailability of quercetin, (a flavonoid, known for its vascular function), is conjugated into glucuronide/sulfate metabolites, before being absorbed, reaching approximately µM levels within a few hours after intake and a half-life of around four hours [193]. quercetin presents a low bioavailability, probably attributed to its poor affinity with the different lipid phases found in the small intestine, inhibiting the uptake into CaCo-2 cells (intestine cells) [194]. However, the quercetin prenylation to 8-prenyl quercetin (8-PQ) is used as a strategy for elevating its lipophilicity and exert anti-inflammatory effects stronger than non-prenylated quercetin in macrophage cells [195].

Recently, new DDSs on the order of nanometers, in the nanometer range, are being engineered to improve the solubility of hydrophobic compounds, minimize systemic side effects and/or enhance the bioaccessibility and bioavailability of nutraceuticals (Table 4). Bioaccessibility is a property that refers to the quantity of a compound that is released from its matrix in the gastrointestinal tract, becoming available for absorption and reaching blood stream. Nanometric delivery systems, derived from food-grade phospholipids and biopolymers, adopt many forms, including liposomes, micelles, micro/nanoemulsions, NPs, polyelectrolyte complexes and hydrogels. The small particle size and the customized materials used to create delivery systems offer some unique properties, such as higher bioaccessibility, stability and resistance to enzymatic activity in the gastrointestinal tract [196]. The polymer NPs, typically assembled from dense proteins and polysaccharides matrix, increase the bioavailability and bioaccessibility of bioactive compounds, due to higher water solubility, with a tendency to increase when mixed with oil droplets. This takes place by promoting solubilization of the bioactive in the micelle phase of the small intestine. Its bioaccessibility depends on bioactive-polymer interactions and susceptibility to digestive enzymes [187]. For example, resveratrol presents a high intestinal absorption (˃70% of the administered dose), but a low oral bioavailability (less than 1–2% of the dose). Calvo-Castro et al. [197] showed a new approach to significantly increase the hydrophilicity and thus, the bioavailability of resveratrol, using a liquid micellar formulation, without any adverse effects. It has been reported that resveratrol in ovalbumin–carboxymethyl cellulose NPs improves the photostability of trans-resveratrol when it is exposed to UV light and releases profile in the in vitro simulated gastrointestinal tract [198]. The development of a novel self-microemulsifying formulation (Capryol 90, Cremophor EL and Labrasol) for codelivery of resveratrol and curcumin, one of the most described nutraceuticals, results in an enhanced oral absorption and an improvement of the poor oral bioavailability of both compounds, which are not very water-soluble [199]. In addition, the interest of curcumin has increased because of its synergistic effects in addition to conventional therapeutic agents for various diseases, especially cancer [200,201]. In ovarian cancer therapy, the toxicity caused by triptolide, a potential anticancer agent, may be reduced by curcumin, due to its antioxidative stress through mPEG-DPPE/calcium phosphate NPs [202].

In animal models of ulcerative colitis (UC), codelivery of conventional drugs related to UC therapy, together with alternative therapeutic molecules or their combinations have been reported. Hyaluronic acid-functionalized polymeric NPs, to direct the specific drug (siCD98) and curcumin have shown anti-inflammatory effects in colonic epithelial cells and macrophages, protecting the mucosal layer and offering a structurally simple platform to be orally administered [203]. In that sense, a pH-sensitive NPs of curcumin-celecoxib combination reduces the overall toxicity and total dose of celecoxib, providing enhanced efficacy for mitigating UC by synergistic action of these two agents [204]. This novel form of carriers could represent a new strategy to deliver drugs directly to target cells in UC therapy.

As it is possible to observe, the use of NPs has been effective to improve the curcumin low systemic bioavailability. In a recent study, the protective effects of curcumin-loaded PLGA NPs, against mono-iodoacetate-induced osteoarthritis in rats, have been reported. The results reveal that curcumin could reverse hypocellularity and structural changes of articular cartilage in animal models of osteoarthritis. However, the increase in cellularity and matrix is more pronounced when it is encapsulated in PLGA [205].

Other nutraceuticals that offer health benefits have been nanoencapsulated to increase delivery, mobility, cellular uptake, bioaccessibility and stability. Carotenoids, widely distributed in fruit and vegetables, induce health beneficial properties mainly through their antioxidant activity, although their bioavailability is often compromised due to incomplete release from the food matrix, poor solubility and degradation during digestion [210]. Yi et al. [211] confirmed that whey protein isolate (WPI) NPs are good carriers for delivering beta-carotene, by means of the homogenization-evaporation method. This is due to the low release profile in gastric fluids and high release profile in intestinal fluids. Additionally, it has been observed that cellular antioxidant activity of beta-carotene improves with WPI-nanoencapsulation in CaCo-2 cells. Functional characteristics (such as antioxidative, antimutagenic, anticarcinogenic, antimicrobial properties) of green tea polyphenols are limited by their sensitivity to factors like temperature, light, pH, oxygen, etc. CH’s NPs, a polysaccharide derived from chitin, can improve the bioaccessibility of tea derived phenols, by opening tight junctions and/or directly being absorbed by epithelial cells via endocytosis [212].

Most studies have failed to show a good bioavailability of many nutraceuticals, but the use of NPs may represent an alternative method to improve the beneficial effects, thus becoming a natural alternative treatment for several diseases.

### 5.2. Toxicity

Most commonly used nutraceuticals are compounds derived from herb food, plants, fruit and vegetables. Widely consumed nutraceuticals include flavonoid, flavonols and polyphenols, such as resveratrol, catechins and quercetin. A small number of these products do have a toxic potential, associated with hepatotoxicity, genotoxicity and mutagenicity [213,214]. In addition, the safety of some nutraceuticals can be compromised via contamination with toxic plants, metals, mycotoxins, pesticides, fertilizers or drug-supplement interactions [215]. Chemical structures of polyphenols could alleviate cytotoxicity induced by NPs through the inhibition of oxidative stress, hydrodynamic size, zeta potential and solubility caused by some NPs, such as the ones derived from zinc oxide (ZnO) [216]. The use of silver NPs (AgNPs) in several dietary supplements, utilized due to their strong antimicrobial properties, may leak out into the food and be consumed, creating severe health risks when reaching the small intestinal epithelium with their surface characteristics altered or dissolved into silver ions, which could alter their subsequent absorption and toxicity [217]. On the contrary, biopolymers, which are used for NP delivery systems, have well-documented biodegradable, biocompatible, mucoadhesive properties, and they do not decrease cellular viability in different cell lines when loaded with bioactive compounds [218].

The safety and beneficial properties on human health of nutraceuticals is well known (Figure 6). The toxicity and bioaccessibility and/or bioavailability could improve with the use of nanoparticle technology. Some NPs could actually cause potential cytotoxicity, the correct choice of nutraceutical-loaded NPs is important to deliver nutraceuticals and represent an alternative or complement to conventional medicine.

## 6. Future Challenges in DDS

The application of nanomedicine represents a huge breakthrough in the above-mentioned fields and assures an encouraging advance in the next decade. Treatments will become more efficient and safer due to the enormous variety of NP design and functionalization. The lists of potential applications progress to the point where the nanocarrier can be customized to best adjust to a certain active ingredient, a specific environment and then provide fitting drug location at the site of action, in a controlled manner. However, it is relevant to mention that NP-based treatments are not perfect and have challenges to conquer. First, the number of polymeric materials currently available for their utilization as DDS is still limited although the R&D has been moved in the last decade, exceeding expectations, from the micro- to the nanosize scale. The ideal adjustment to the delivery conditions, such as transportation to the site of action, specific targeting or adequate delivery profile, among others, for each type of disease, requires the development of new polymers that can fit these requisites. Although selective targeting supposed a great improvement in comparison to non-encapsulated drugs, it is a very complex mechanism and represents a challenge itself. Overexpression of a specific surface protein is not enough to assure selective targeting as they are also normally expressed in normal cells. This point is more critical in cancer treatments, where administered drugs usually possess higher toxicity that could lead to numerous undesirable secondary effects compared to drugs used in other diseases treatments. Most the assays have been developed in small animal models showing promising results, but the translation from animal results into clinical success has been limited. More clinical research and data are needed to fully comprehend the mechanism of these nanocarriers. In addition, limitations include the uncertain future of pharmaceutical companies which face high expenses concerning clinical trials and decreasing success rates in the flow of novel entities in the R&D pipeline. Examples of polymeric NPs that do not fulfil all the regulatory requirements for clinical evaluations and which had a harmful economic impact for their pharmaceutical companies are Livatag, PACA nanoparticulate formulation containing doxorubicin and BIND-014, PLGA polymer conjugated to docetaxel [219]. These formulations were potentially useful for the treatment of hepatocellular carcinoma and prostate cancer, respectively [219]. While BIND-014 began phase II of the clinical trial in 2018, the phase III studies of Livatag have not meet its primary endpoint of improving survival over, although it’s action mechanism was demonstrated through DNA damage/synthesis inhibition and a decrease efflux pump by *P*-gp, due, at least in part, by an ion-pair association of doxorubicin with soluble degradation products of PACA which, conversely to free doxorubicin, are not a substrate for *P*-gp [220]. However, in both cases, no improvement was found when evaluated [221,222,223]. Perhaps focusing on more specific diseases, also considering aging population, novel formulations or indications for previous blockbusters drugs, including polymeric NPs, could be a good recommendation to maintain a profitable economic growth rate. Achieving reasonable success for oral bioavailability of poorly absorbed lipophilic and hydrophobic drugs, to maintain adequate and effective plasma levels over prolonged periods of time, still remains an important challenge. In addition, the fact that drugs used for severe illness are usually administered only through the parenteral route and the inaccessibility of most pharmacological targets are major constraints that are increasing interest in developing more efficient nanodelivery systems. Conceiving new methods for the manufacture of NPs at reasonable costs is an important part of this challenge because there are only a small number of them that fulfill the appropriated requirements to reach the target and to subsequently deliver the drug in a suitable manner. It is also mandatory for these polymeric NPs to be biodegradable or to possess a high capacity to be eliminated outside the body avoiding accumulation, being nontoxic and non-immunogenic. It is remarkable to point out the role that copolymers could play in tuning or modulating the interactions with mucosa or blood proteins in order to control their in vivo fate or to stabilize NPs without the need of surfactants entities. It would be also interesting for the near future research in this field to include stimuli responding polymers which can confer triggered release properties. From a manufacturing point of view, nanospheres and nanocapsules could be easily obtained by applying the existing methods, but new structures like polymersomes, are still waiting for better synthesis to join the family of nanoparticulate DDS. The need for developing NPs with many capabilities (targeting, image contrast enhancement), named as multifunctional NPs, means more synthetical steps, more regulatory hurdles and higher expenses. Conquering these objectives may seem very difficult, but there is hope of reaching a better scenario.

Over the last few years, there has been a global transformation in the field of nanomedicine, which has led to a multidisciplinary and collaborative approach with promising results and success. The future path of collaborations between theoretical and experimental scientists as well as the pharmaceutical industry, physicians and the regulatory agencies, will be crucial and will allow us to implement the laboratory results into the clinic and therefore, initiate the next generation of clinical therapies, trying to minimize the devastating consequences of terrible diseases such as pandemic covid-19.

In conclusion, many drawbacks or limitations still need to be resolved through numerous efforts and concentrated interdisciplinary scientific collaboration in order to reach the desired goals.

## 7. Conclusions

The toxicity associated with certain drugs and classical formulations or the complexity of treatment of some diseases, have driven the development of new alternatives as DDS. Among these, polymeric NPs are gaining high attention due to the biocompatibility, biodegradability and versatility they can offer, opening a wide range of materials that could possess the required characteristics for a specific application. For example, the use of hyaluronic acid in the NP outer surface increases adhesion to mucosal tissue and hence active ingredient liberation time, which is beneficial for drug delivery to eyes. Different techniques for cancer diagnosis are used with some disadvantages, such as the difficulty for early stage detection. The optimization of these techniques is possible due to different types of contrast agents, being NPs (e.g., gadolinium-based materials or AuNP) a promising agent in medical applications by the excellent biocompatibility, good water solubility and low toxicity. NP protection with PEG increases magnetic nanomaterials stability and avoids recognition by macrophages, which increases circulation time, which is indeed a requirement for diagnosis. At the same time, given that ABC transporters mediated MDR is the main obstacle for effective cancer therapy, the use of PEG as coating material for polymeric NPs has recently been described as an effective tool for inhibiting ABC transporters. The simultaneous use of one single NP for both cancer detection and drug delivery makes NPs a potential theranostic. Regarding NP pathways for drug delivery, passive diffusion, active targeting as well as stimuli responsive systems have been described. In this respect, the functionalization of NPs with the precise antibody, improves recognition of the specific site of action to achieve therapeutic effect, which drastically reduces secondary effects of drugs for oncologic treatments. In addition, the inclusion of highly unstable compounds used as nutraceuticals inside PVs, prevents them from being exposed to environments that could affect their integrity, implying an improvement in their absorption by the gastrointestinal system and hence, an increment of their bioavailability. This would suggest a new approach in nanomedicine for the use of nutraceuticals as an alternative or complementary treatment for different pathologies. Although important progress has been made in the fields of ocular drug delivery, cancer diagnosis and treatment and nutraceutical delivery, areas of medicine with an associated high level of morbidity, a notable reduction in the patient’s quality of life or even an important mortality, in most cases, the translation from animal tests to real clinical success has been limited. The efforts applied in the development of new polymeric materials that may encompass the specific requirements for a certain delivery system, the better knowledge the scientists have about disease mechanisms and the collaborative research work carried out among all scientific areas, will boost the current state of the use of NPs in the medical field, which will be translated into more efficient and safer treatments.

## Figures and Tables

**Table 1:** Polymeric micelles used for drug delivery, drug encapsulated and disease for which they are administered.

Type of Nanoparticle	Nanoparticle Composition	Drug Delivery	Treatment	Reference
Polymeric Micelles	PLGA/PVA	bevacizumab	Choroidal and retinal neovascularization	[33]
		dexamethasone	Ocular inflammation	[34]
		fenofibrate	Retinal dysfunctions, retinal leukostasis, retinal vascular leakage, over expression of VEGF, choroidal neovascularization	[35]
	PLGA/PVA/PEI	bevacizumab and dexamethasone	Choroidal neovascularization	[36]
	PLGA/Tween 80, poloxamer 188 or Brij^®^	brinzolamide	Ocular pressure	[37]
	PLGA/Pluronic F127	dexamethasone	Immunologic graft rejection	[38]
	PLGA/PVP	bevacizumab	Age-related macular degeneration	[39]
	CH/Sodium tripolyphosphate	levofloxacin	Ocular infections	[40]
		bevacizumab	Choroidal neovascularization	[41]
	CH/Sodium tripolyphosphate/hyaluronic acid	ceftazidime	Ocular infections	[42]
	CH/PVA/sodium deoxycholate	prednisolone	Ocular inflammation	[43]
	Stearic acid and valylvaline functionalized CH	dexamethasone	Ocular inflammation, retinal dysfunctions, retinal leukostasis, retinal vascular leakage, over expression of VEGF, choroidal neovascularization	[44]
	Cationic CH grafted methoxy poly(ethylene glycol)-poly(ε-caprolactone)	diclofenac	Ocular inflammation	[45]
	Methoxy poly(ethylene glycol)-poly(lactide) block copolymer	cyclosporine A	Dry eye syndrome	[46]
	Tween80/polyoxyethylene stearate	everolimus	Autoimmune uveoretinitis, non-infectious uveitis, corneal neovascularization and immune-mediated rejection	[47]
	PVA/Poloxamer P407/hydroxypropyl methylcellulose			
	PEG–PCL–PEG	triamcinolone acetonide	Ocular inflammation	[48]
	Lecithin-based NPs embedded in poloxamers gel (P188 and P407)	dexamethasone		[49]
	PLGA–PEG NPs embedded in PEG–PLGA–PEG gel	triamcinolone acetonide	Age-related macular degeneration	[50]
	Bevacizumab-coated PLA NPs embedded in PLGA microparticles	bevacizumab		[51]
Dendrimeric nanocarriers	PEGylated polyamidoamine modified with cyclic arginine–glycine–aspartate hexapeptide and penetration	–	Posterior ocular diseases	[52]
	Timolol-derivatized polyamidoamine	timolol	Ocular hypertension	[53]
	Polyamidoamine/hyaluronic acid	antisense oligonucleotides	Regulation of the expression of target proteins and genes in cells	[54]
Cyclodextrins	Propylamino-β-Cyclodextrin	latanoprost	Glaucoma	[60]
	γ-Cyclodextrin and randomly methylated β-cyclodextrin	celecoxib	Age-related macular degeneration and diabetic retinopathy	[61,62]
	α-Cyclodextrin/Soluplus/Pluronic P103	natamycin	Fungal keratitis	[63]
Polymeric vesicles	DOTAP/DOPE/DSPE–PEG	siRNA sequences/chlorhexidine	Keratitis caused by *Acanthamoeba*	[65]
	Precirol^®^ ATO 5/castor oil/Span^®^ 80/mPEG-2K-DSPE	natamycin	Fungal keratitis	[66]

PLGA—poly(lactic-co-glycolic acid); PVA—poly(vinyl alcohol); VEGF—Vascular Endothelial Growth Factor; PEI—poly(ethyleneimine); PVP—poly(vinylpyrrolidone); CH—chitosan; PCL—poly(ε-caprolactone); PEG—poly(ethylene glycol); DOTAP—1,2-dioleoylsn-glycero-3-trimethylammonium propane; DOPE—1,2-di-(9E-octadecenoyl)-sn-glycero-3-phosphoethanolamine; DSPE–PEG—1,2-distearoyl-sn-glycero-3-phosphoethanolamine-*N*-[methoxy(polyethylene glycol)-2000]; PEG-2K-DSP—*N*-(Carbonylmethoxypolyethylenglycol-2000)-1,2-distearoyl-sn-glycero-3-phosphoethanolamine.

**Table 2:** Anticancer drug polymer nanoparticles organized by cancer type they are used for.

Polymer	Active Principle	Type of Cancer	Experimental Model/Route	Size (nm)	Z Potential (mV)	PDI	References
PEGylated PLGA	doxorubicin	various	In vivo: Bioavailability assay in Wistar rat Oral	183.10	−13.10	0.132	[136]
PACA	doxorubicin–cyclosporin A.	various	In vitro: P388/ADR cells line	288	*	*	[137]
Lip–BSA	paclitaxel	various	In vivo: 4T1 cells in BALB/c mice Tail vein	116.2	−18.4	0.307	[138]
PCL–PEG	camptothecin	glioma	In vivo: 4T1 cells in BALB/c mice Tail vein	274	−19	0.07	[139]
PLGA–PEG	paclitaxel	glioma	In vivo: gliosarcoma 9L cells in Fischer F344 rats Direct injection	121	23.7	0.088	[140]
PLGA–Cyanine5.5	doxorubicin	glioblastoma	In vivo: C6 Glioma cells in Wistar rats and nude mice Tail vein	114	−14.9	0.196	[141]
PBCA	doxorubicin	glioblastoma	In vitro: U87 glioblastoma human cells line	260	−19	0.02	[142]
PLA–PEG–maleimide	paclitaxel	breast cancer (TNB)	In vitro: MDA-MB-231 cells In vivo: BALB/c homozygous nude mice Intravenous injection. Tail vein	212	−16.34	0.183	[143]
mPEG–PLGA–PGlu	doxorubicin–curcumin	breast cancer	In vivo: LM2 cells in BALB/c homozygous nude mice Tail vein	107.5	−13.7	*	[144]
PCLLA–PEG–PCLLA	doxorubicin and Chlorin e6-MnO_2_	breast cancer	In vivo: MCF-7/ADR cells xenograft in female BALB/c nude mice. Tail vein	120	−8.9	*	[145]
TPGS–PLGA	doxorubicin and metformin	breast cancer	In vivo: MCF-7 cells in nude mice Tail vein	87	−3.5	0.5	[146]
TPGS–PLGA	docetaxel and salinomycin	breast cancer	In vitro: MCF-7/DOX cell line	73.83	−25.7	0.193	[147]
Gal–pD–TPGS–PLA	docetaxel	liver cancer	In vivo: MCF-7 cells in BALB/c mice Orthotopic injection	209.4	13.7	0.145	[149]
PCL–PEGPEG–PCL	paclitaxel	lung cancer	In vivo: MCF-7/ADR cells in BALB/c nude mice Intravenous injection	168	−12.49	0.19	[150]
PEI–PLA	paclitaxel	lung cancer	In vivo: A549 cells in BALB/c mice. Tail vein	67.31	30.3	0.105	[151]

PCLLA—poly(caprolactone-co-lactide); PBCA—poly(butylcyanoacrylate). * Data not included in the corresponding publication.

**Table 3:** Inhalable polymeric nanocarriers.

Polymeric Nanocarrier	Active Principle	Preparation Method	Size (nm)	Z Potential (mV)	PDI	References
Gelatin NPs	cisplatin	desolvation	220	−9.3	0.287	[152,153,154]
HSA NPs	doxorubicin + TRAIL	self-assembly	341.6	*	*	[155]
CH/PLGA NPs	OMR	emulsion–diffusion	160	29	0.033	[156,157]
BIPCA NPs	doxorubicin	emulsion polymerization	137.2	23.5	0.12	[158]
PEGylated PAMAM dendrimers	doxorubicin	chemical conjugation	26.1	−6.6	0.108	[159]
Hyaluronan conjugates	cisplatin	covalent bonding	*	*	*	[160]
PEI polyplexes	p53	electrostatic complexation	*	*	*	[161]
SDA–PEI polyplexes	PDCD4 + shAkt1	electrostatic complexation	*	*	*	[162]
Glucosylated PEI polyplexes	PTEN	electrostatic complexation	*	*	*	[163]
UACH polyplexes	PDCD4 PTEN	electrostatic complexation	*	*	*	[164,165]
SPE–GPT polyplexes	shAkt1	electrostatic complexation	163.2	9.14	0.192	[166]
SPE–PEG polyplexes	PDCD4	electrostatic complexation	130	8.61	1.13	[167]
CH–g–PEI polyplexes	shAkt1	electrostatic complexation	166.4	−20	*	[168,169]
PLL/protamine polyplexes	p53	electrostatic complexation	*	*	*	[170]
PEI–alt–PEG polyplexes	Akt1 siRNA	electrostatic complexation	*	*	*	[171]
PEI polyplexes	doxorubicin + Bcl2 siRNA	electrostatic complexation	78.2	20.4	*	[172,173]

HSA—human serum albumin; TRAIL—tumor necrosis factor-related apoptosis-inducing ligand; OMR—antisense oligonucleotide 2′-O-methyl-RNA; BIPCA—poly(isobutyl cyanoacrylate); PAMAM—poly(amidoamine); p53—tumor suppressor gene; SDA—sorbitol diacrylate; PDCD4—cDNA of programmed cell death protein 4; PTEN—phosphatase and tensin homolog deleted on chromosome 10 gene; UACH—urocanic acid–modified chitosan; SPE–PEG—spermine-*alt*-poly(ethylene glycol) polyspermine; shAkt1—hRNA-silencing Akt1. * Data not included in the corresponding publication.

**Table 4:** Development and use of nutraceutical-loaded polymeric nanoparticles on different pathologies.

Drug Delivery	Polymeric Nanoparticle	Experimental Model/Route	Results	Reference
CD98 siRNA plus curcumin	HA-functionalized NP encapsulated in hydrogel (CH: alginate; 3:7)	In vitro: Caco2-BBE and Raw 2647 cells	↑Cellular uptake ↓Expressions of CD98 and TNF-α	[203]
		In vivo: DSS-induced UC/orally	↓Weight loss ↓Fecal Lcn-2 levels ↓MPO activity ↓Histological damage ↓CD98 and TNF-α mRNA expression	
curcumin plus celecoxib	pH sensitive enteric polymer NP (Eudragit^®^ S100)	In vivo: TNBS-induced UC/orally	↓MPO, SOD and LPO ↓Leukocyte infiltration	[204]
curcumin	Biopolymeric CH NP	In vitro: HeLa cells	↓Proliferation and viability cell ↑Apoptotic activity, DNA damage, cell-cycle blockage and ROS levels	[200]
curcumin	PLGA NP	In vivo: MIA-induced OA/orally	↑Cellularity and matrix	[205]
curcumin	Theracurmin^®^ NP	In vivo: DSS-induced UC/orally	↓NF-κB, TNF-α, IL-1β, IL-6, CXCL1 and CXCL2 and neutrophil infiltration ↑CD4+ and Foxp3+ T cells ↑CD103+ and CD8α− dendritic cells ↑*Clostridium* cluster IV and XIVa ↑Butyrate levels (bacteria and fecal)	[206]
resveratrol plus quercetin	PEG modified CH NP	Ex-vivo: Albino rabbit cornea	↑Solubility and permeation ↓Intraocular pressure	[207]
docetaxel plus resveratrol	EGF conjugated core-shell lipid–polymer hybrid NP	In vitro: HCC827, NCIH2135 and HUVEC cells	↓Tumoral cell viability	[208]
		In vivo: lung cancer animal model/intravenously	↓Body weight loss ↓Tumor volume ↑Tumor growth inhibition	
resveratrol	Galactosylated NP (NP combined with a ligand (galactose) for improved route of intestinal transport by the way of SGLT1)	In vitro: Raw 2647 cells	↓TNF-α, IL-6 and NO	[209]

↑: increase; ↓: decrease; CMC—carboxymethylcellulose; CXCL 1 and 2—chemokine ligand -1 and 2; DSS—dextran sulfate sodium; EGF—epidermal growth factor; HA—hyaluronic acid; IL—interleukin; Lcn.2—lipocalin-2;LPO—lipid peroxidation; MIA—mono-iodoacetate; MPO—myeloperoxidase; NO—nitric oxide; OA—osteoarthritis; OVA—ovalbumin; ROS—reactive oxidative species; SOD—superoxide dismutase; TNBS—trinitrobenzene sulfonic acid; TNF-α—tumoral necrosis factor-α; UC—ulcerative colitis; SGLT1—sodium glucose–linked transporter 1.
